# An Evaluation of Trainee-Executed Ultrasound-Guided Nerve Blocks in the Emergency Department

**DOI:** 10.3390/jcm15187121

**Published:** 2026-09-14

**Authors:** Travis P Martin, David Wasiak, Tyler Jackson, Eric Kersjes, Omar G Rizvi, Jonathan Coss, Richard Amini, Steven Haight, Josie Acuña, Srikar Adhikari

**Affiliations:** 1 College of Medicine, University of Arizona, Tucson, AZ 85724, USA; tmartin@aemrc.arizona.edu (T.P.M.); dwasiak@aemrc.arizona.edu (D.W.); tylerpjackson@arizona.edu (T.J.); ekersjes@aemrc.arizona.edu (E.K.); omarrizvi@arizona.edu (O.G.R.); jcoss@arizona.edu (J.C.); jacuna1@arizona.edu (J.A.); sadhikari@arizona.edu (S.A.); 2 Department of Emergency Medicine, University of California, Fresno, CA 93701, USA; stephenhaight@gmail.com

**Keywords:** ultrasound, ultrasonography, emergency medicine, nerve block, regional anesthesia, education, point-of-care

## Abstract

**Background/Objectives**: Peripheral nerve blocks offer the option of targeted pain relief localized to the injured areas while minimizing potential systemic side effects of opioids such as hypotension and respiratory depression. However, there is often hesitancy in the acute setting to perform these procedures. The objective of this study was to describe the technical performance and recorded complications of archived ultrasound-guided and ultrasound-assisted peripheral nerve blocks performed by Emergency Medicine trainees in the emergency department (ED). **Methods**: A retrospective review of patients presenting at two urban academic EDs who underwent ultrasound-guided peripheral nerve blocks. Data were collected from the ED ultrasound image archiving system and electronic medical record and included age, gender, level of provider training, and ultrasound image data. The primary outcome was technical performance, including needle-tip visualization and visualization of anesthetic infiltration. Secondary outcomes included recorded complications and technical outcomes by level of trainee experience. **Results**: A total of 72 patients were included in the final analysis. Ulnar nerve blocks were the most common procedure performed at 33%. No major complications were documented in the available records; one patient had documented paresthesia. On ultrasound image review, the needle tip was clearly visualized in 47/72 (65%) of blocks and infiltration of anesthetic was seen in real time in 79% of subjects. **Conclusions**: Among archived trainee-performed ultrasound-guided peripheral nerve blocks, needle-tip visualization and anesthetic-infiltration visualization varied across procedures and levels of training, and few complications were recorded. These findings provide descriptive data regarding the technical performance of trainee-performed ultrasound-guided nerve blocks but should not be interpreted as establishing their overall safety or clinical effectiveness.

## 1. Introduction

Acute pain is one of the most common presenting complaints in the emergency department (ED) [1]. Managing the pain of patients with extremity fractures, lacerations, and burns can be difficult while managing the demands of a busy ED, as well as finding appropriate levels of analgesia while minimizing side effects. The current opioid epidemic and the associated side effects and risks with opioid medications, especially in certain populations, further complicate pain management in the ED. Approximately 42% of opioids prescribed from the ED may ultimately be misused. Of the opioid prescriptions that are diverted, approximately 10% originate from an ED prescription. Among patients who suffer an opioid-related death, approximately 1.8% of the opioid pain reliever pills given to the decedents will have come from the ED [2].

Effective pain management in the ED requires balancing adequate analgesia with the potential adverse effects of systemic analgesic medications. Peripheral nerve blocks may serve as one component of a multimodal analgesic approach by providing targeted regional analgesia while minimizing systemic opioid exposure and associated adverse effects such as hypotension and respiratory depression [3]. Prior studies have demonstrated that emergency physicians utilizing nerve blocks of the extremities is a safe and effective method that can be used for pain management and results in high levels of satisfaction among both patients and physicians [4,5]. The increasing availability of point-of-care ultrasound in the ED provides an opportunity to incorporate these regional anesthesia techniques into the management of acute pain. Despite the potential benefits of regional analgesia, some emergency physicians may be hesitant to perform peripheral nerve blocks because of limited training and procedural experience with these techniques. Ultrasound-guided nerve blocks require familiarity with relevant sonoanatomy as well as technical skills such as needle visualization and real-time needle guidance.

The objective of this study was to describe the technical performance and recorded complications of archived ultrasound-guided and ultrasound-assisted peripheral nerve blocks performed by Emergency Medicine trainees in the ED.

## 2. Materials and Methods

### 2.1. Study Design and Setting

This study consisted of a retrospective review of patients who received an ED ultrasound-guided nerve block by ED residents or fellows. This study includes ED nerve blocks performed at two urban academic EDs, with visits totaling approximately 110,000 patient visits per year. Both EDs have an ACGME (Accreditation Council for Graduate Medical Education)-accredited Emergency Medicine residency program. One ED has an additional five-year combined Emergency Medicine/Pediatrics residency program. The program also has an Emergency Ultrasound fellowship-training program, with fellows working as attendings and scanning shifts at both sites. Exclusion criteria included the inability to determine if a fellow or resident completed the block, or if the ultrasound images of the block were not archived in the ultrasound database. Approval from the University of Arizona Institutional Review Board was obtained for this study.

Residents performing blocks in this study receive ultrasound training per ACGME guidelines [6]. In their first year of training, all residents complete a 2-week rotation in Emergency Ultrasound where they receive didactics and hands-on experience in basic ultrasound technique. Emergency Medicine residents also received quarterly procedure labs utilizing point-of-care ultrasound (POCUS) and needle visualization. The training sessions were approximately 1–2 h long and taught by Emergency Ultrasound fellows or Emergency Ultrasound fellowship-trained faculty. The residents’ first sessions took place in their first year of residency. They were given an introductory lecture highlighting the technique and benefits of ultrasound for nerve blocks. Residents were walked through a basic protocol for performing the procedure, including image optimization using controls such as focus, gain, and depth. Both ultrasound-guided and ultrasound-assisted techniques were discussed, but residents were encouraged to perform ultrasound-guided procedures when possible. Training progressed from basic ultrasound skills, including identification of relevant sonoanatomy and image optimization, to needle visualization and real-time ultrasound-guided needle advancement. Residents practiced these skills during dedicated ultrasound rotations and procedural training sessions before applying them clinically under attending supervision. The specific peripheral nerve block performed in the ED was determined by the clinical indication rather than by a predefined sequence of block types.

Residents were given a hands-on session practicing needle guidance for instances in which the ultrasound-guided technique was used. Needle guidance and enhancement software were not available or provided on the software at either site or in the ED. Emphasis was placed on enhancing needle visualization with gain and depth settings, as training in these skills has been shown to assist in overall procedure success in trainee settings [7,8,9]. The in-plane needle approach was strongly encouraged to keep the entire needle in view to ensure safety of the procedure. A variety of standard, cart-based ultrasound systems were available for use in training sessions and in the ED. These included the Mindray M-9 (Shenzhen, China), Philips CX50 (Amsterdam, The Netherlands), Zonare Ultra (Zonare Medical Systems, Mountain View, CA, USA), and Philips Sparq (Philips Healthcare, Andover, MA, USA).

In the study, the decision to perform the nerve block was ultimately at the discretion of the treating Emergency Medicine attending physician, with direct supervision and assistance provided as needed based on the resident’s experience level and patient acuity. Residents were instructed to save all POCUS examinations to a web-based workflow solutions database, Q-Path (Q-path, Telexy Healthcare, Maple Ridge, BC, Canada), so that all studies could undergo quality assurance by either Emergency Ultrasound fellows or Emergency Ultrasound fellowship-trained faculty. The primary outcome was technical performance of archived ultrasound-guided and ultrasound-assisted peripheral nerve blocks, including needle-tip visualization and visualization of anesthetic infiltration. Secondary outcomes included recorded complications related to the procedure and technical outcomes stratified by level of trainee experience.

### 2.2. Study Protocol

The Q-Path database was initially queried for eligible subjects who received ultrasound-guided nerve blocks in the ED, followed by an electronic medical record (EMR) review. The database complies with relevant data protection and privacy regulations. A chart reviewer performed data abstraction using a standardized data extraction form. The chart reviewer first confirmed whether the nerve block was performed by a resident and documented their year in training (PGY-1/PGY-2/PGY-3, etc.). Data included age, gender, level of provider training, and ultrasound image data, including visibility of the peripheral nerve, needle-tip visualization, and visualization of anesthetic infiltration.

Evaluation of POCUS procedures was supervised by a trained attending who determined image quality and appropriate anatomy. At both institutions, it is standard that attending physicians be physically present for the key components of a resident-performed procedure. During the procedure, appropriate images and video clips of the target anatomy, needle, and anesthetic delivery were visualized per training protocols. The resident and attending would then fill out worksheets in Qpath describing the procedure and indications, as well as additional labeling of imaging. Finally, for the purpose of this study, uploaded images were analyzed by Emergency Ultrasound faculty and fellows.

All archived images were reviewed by an Emergency Ultrasound fellow who was blinded to the study hypothesis and outcomes. The American College of Emergency Physicians (ACEP) clinical guidelines for ultrasound imaging and the associated ACEP literature specific to performing nerve blocks were used to create the basic criteria for this study [10,11]. Procedure-related complications were identified through a retrospective review of the available electronic medical record. Complications were recorded when documented during the ED encounter or in available subsequent clinical documentation. No standardized prospective follow-up protocol was used to assess for delayed complications. Study data were collected and managed using REDCap electronic data capture tools [12,13].

### 2.3. Data Analysis

Descriptive statistics were used to summarize the data. Continuous data were presented as means and categorical data were presented as frequencies and percentages with 95% confidence intervals.

## 3. Results

Ultrasound was utilized in all 74 procedures. Of these, 69 (93.2%) were performed using an ultrasound-guided technique with real-time needle guidance, while 5 (6.8%) were performed using an ultrasound-assisted technique. Of the 74 procedures, 72 (97.3%) were performed by residents and 2 (2.7%) were performed by fellows. Because the primary analysis focused on resident-performed procedures, the 72 resident-performed blocks comprised the final analytic cohort. Of the 72 procedures included in the final analysis, 48 (66.7%) were performed in male patients and 24 (33.3%) in female patients. The mean patient age was 41.0 years (SD, 22.6).

**Figure 1:**
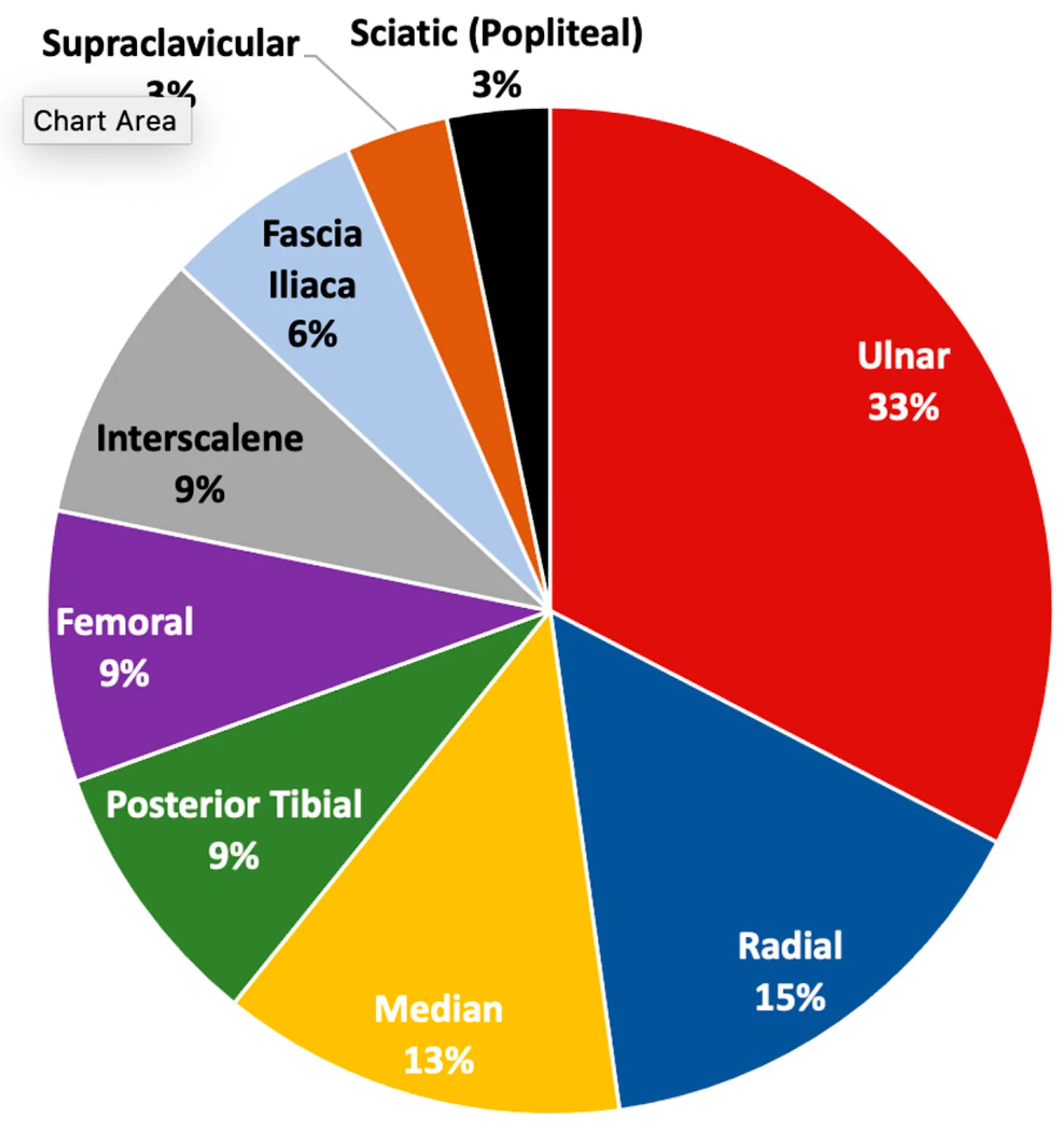
Distribution of ultrasound-guided and ultrasound-assisted peripheral nerve blocks.

Data were obtained over a 2-year period. The ultrasound-guided nerve blocks were performed by residents at various levels of training. Of the 72 resident-performed nerve blocks included in the final analysis, the postgraduate year was available for 58 procedures: 20 were performed by PGY-1 residents, 12 by PGY-2 residents, and 26 by PGY-3 residents. PGY level was unavailable for the remaining 14 procedures. Analyses stratified by training level were therefore limited to the 58 procedures with documented PGY levels. The different types of ultrasound-guided nerve blocks performed are summarized in Figure 1. Ulnar nerve blocks were the most common procedure performed at 33%.

**Figure 2:**
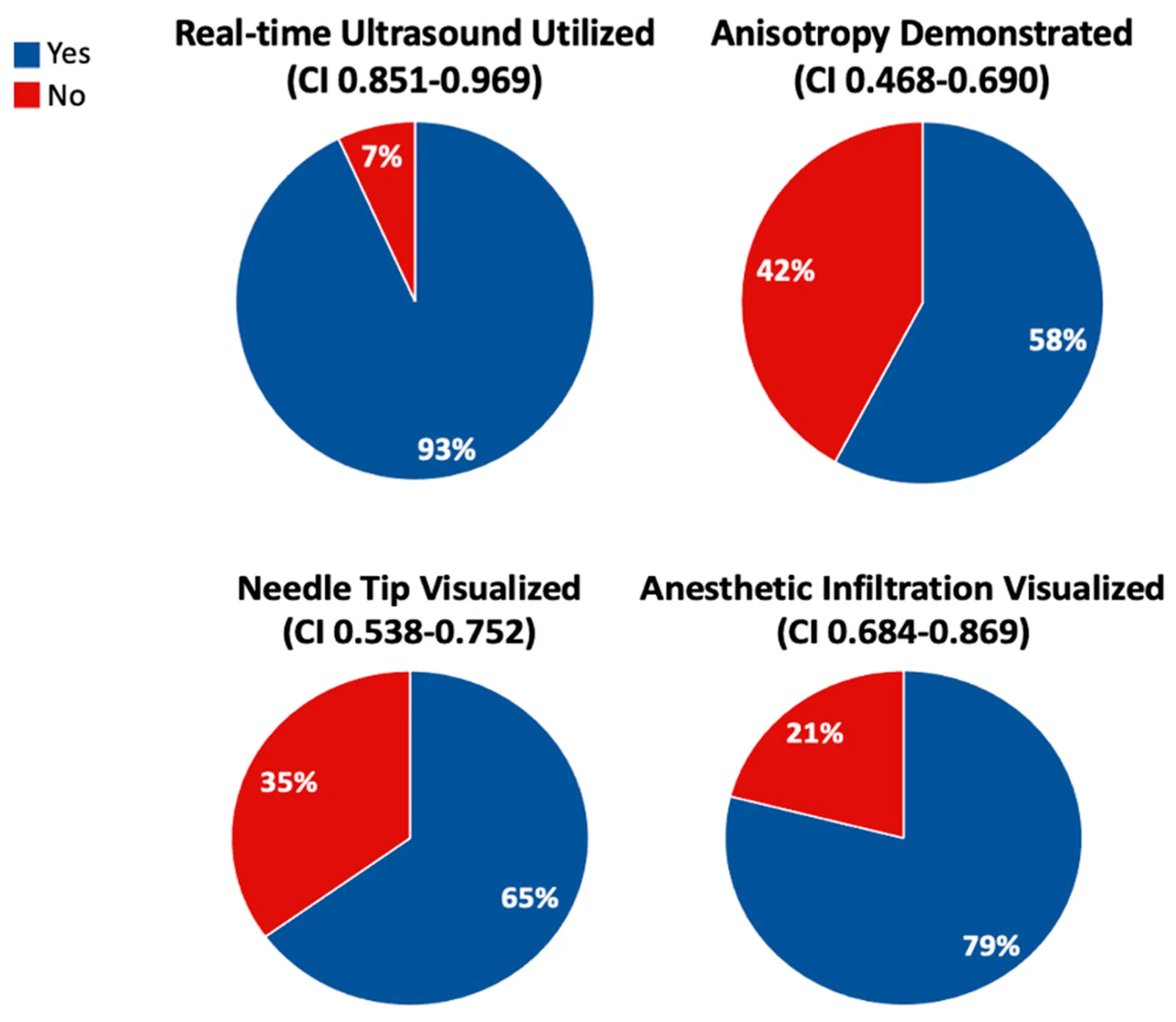
Ultrasound guidance technique-related outcomes.

The needle tip was clearly visualized in 47 of 72 procedures (65.3%; 95% CI, 53.8–75.3%), and real-time anesthetic infiltration was visualized in 57 of 72 procedures (79.2%; 95% CI, 68.3–87.0%). Ultrasound guidance was utilized in 69 of 74 procedures (93.2%; 95% CI, 87.0–98.0%), and 72 of 74 nerve blocks (97.3%; 95% CI, 90.7–99.3%) were performed by residents. One patient experienced paresthesia after receiving an ulnar nerve block, representing the only recorded complication in the archived cohort (Figure 2).

## 4. Discussion

In recent studies, it is estimated that chief complaints involving pain amount to roughly 70–80% of all patient encounters in the ED [14]. As clinicians in an acute care setting, identifying the source and ruling out critical diagnoses for pain-based symptoms is a top priority. Yet, despite the frequency of pain-based symptoms, oligoanalgesia continues to be a common, well-documented occurrence in Emergency Medicine [15,16,17]. This is compounded by the rising number of ED visits, an ongoing national opioid crisis creating increased scrutiny on pharmaceutical intervention, and continued strain on ED staffing, all of which have created a critical need for reliable, long-term analgesia. For many physicians in the acute care setting, the use of regional anesthesia has become a potential solution to consider.

The use and safety of regional anesthesia are well documented in the anesthesia literature and have been utilized for years for numerous inpatient and surgical indications. In the last 10–20 years, its use has also increased in the ED setting. Whether used as an adjunct for traumatic injuries such as fractures and lacerations or as part of a multimodal pain management strategy, regional anesthesia can provide prolonged sensory analgesia, with or without motor blockade, and may reduce the need for systemic opioid analgesia [18,19]. The ability to provide targeted analgesia may be particularly advantageous in the acute care setting, where clinicians must balance effective pain control with the potential adverse effects of systemic medications. In addition, peripheral nerve blocks can complement rather than replace other analgesic modalities, allowing them to be incorporated into an individualized multimodal approach to pain management. These characteristics make peripheral nerve blocks a potentially valuable component of multimodal acute pain management.

Traditionally, peripheral nerve block techniques were performed using fluoroscopy, nerve stimulation, or landmark-based methods, often by anesthesiologists or proceduralists trained in interventional pain management or regional anesthesia. With the advent and increasing availability of point-of-care ultrasound (POCUS), ultrasound-guided peripheral nerve blocks have become increasingly accessible to emergency physicians. Ultrasound allows clinicians to directly identify relevant anatomic structures and visualize the relationship between the needle, target nerve, and surrounding vascular and soft-tissue structures during the procedure. These capabilities are particularly relevant in the ED, where regional anesthesia may provide a targeted component of multimodal pain management while limiting reliance on systemic analgesic medications. At the same time, successful incorporation of these techniques into emergency practice requires competency in sonoanatomy, image optimization, needle visualization, and real-time needle guidance. Developing and maintaining these skills may be challenging when individual nerve block procedures are encountered infrequently during residency training. While POCUS as an adjunct for procedural skills and needle visualization has been a core Emergency Medicine residency requirement since 2013, some physicians may remain hesitant to learn or attempt these procedures because of limited training or procedural experience [6]. Dedicated ultrasound rotations, procedural laboratories, simulation, and supervised clinical practice may therefore provide opportunities for trainees to develop these technical skills before independent practice. The findings of this study provide descriptive data regarding the technical performance and recorded complications of ultrasound-guided peripheral nerve blocks performed by Emergency Medicine trainees with varying levels of experience.

Currently, specialty-wide training and accreditation criteria specific to regional anesthesia do not exist. However, a wide range of ultrasound-guided procedures are incorporated into the Accreditation Council for Graduate Medical Education (ACGME) core training requirements for Emergency Medicine residents and are also addressed in the ACEP ultrasound guidelines. Using these recommendations as a framework, residents at our institutions received longitudinal POCUS education throughout residency and were encouraged to incorporate ultrasound into their clinical practice during ED shifts. In addition, regular conference didactics and annual simulation laboratories provided opportunities for continued training and supervised skills practice. This longitudinal approach allowed residents to develop foundational ultrasound competencies, including image acquisition, image optimization, and recognition of relevant anatomy, before applying these skills to more technically demanding ultrasound-guided procedures. For peripheral nerve blocks specifically, the ability to identify the target nerve and surrounding structures must be combined with procedural skills such as needle visualization and real-time needle guidance. Repeated exposure and supervised practice may therefore be particularly important as residents progress from acquiring basic POCUS skills to integrating ultrasound guidance into procedural care. All Emergency Medicine residents at our institutions were also scheduled for a dedicated 2-week ultrasound rotation and could select additional ultrasound training as part of their annual elective rotations. This educational framework represented the baseline ultrasound training available to residents performing nerve blocks during the study period.

Needle-tip visualization was selected as an important measure of technical performance because continuous visualization of the injection needle may facilitate appropriate needle placement and minimize unintended contact with vascular, neural, and other soft-tissue structures [20,21]. Needle-tip visualization can be technically challenging, particularly among less experienced operators. In this cohort, the needle tip was visualized in 65.3% of archived procedures, while anesthetic infiltration was visualized in 79.2%. These findings should be interpreted as measures of documented technical performance rather than surrogate measures of procedural safety. Furthermore, needle-tip visualization may have been underestimated because operators may not have saved representative images or clips demonstrating the complete needle trajectory.

In this cohort of archived trainee-performed nerve blocks, only one complication, paresthesia, was documented. However, the retrospective design and reliance on archived procedures limit the ability to determine the true complication rate, as complications associated with undocumented, aborted, or unarchived procedures may not have been captured. Accordingly, these findings should be interpreted as recorded complications within the available cohort rather than evidence establishing the safety of trainee-performed peripheral nerve blocks. One episode of paresthesia was documented following a nerve block in a patient with a shoulder dislocation who subsequently required procedural sedation for reduction. Given the retrospective study design, the relationship between the paresthesia and the nerve block could not be determined. Furthermore, incomplete longitudinal follow-up limits assessment of delayed complications. Prospective studies with standardized follow-up are needed to more accurately characterize complications associated with trainee-performed nerve blocks.

**Figure 3:**
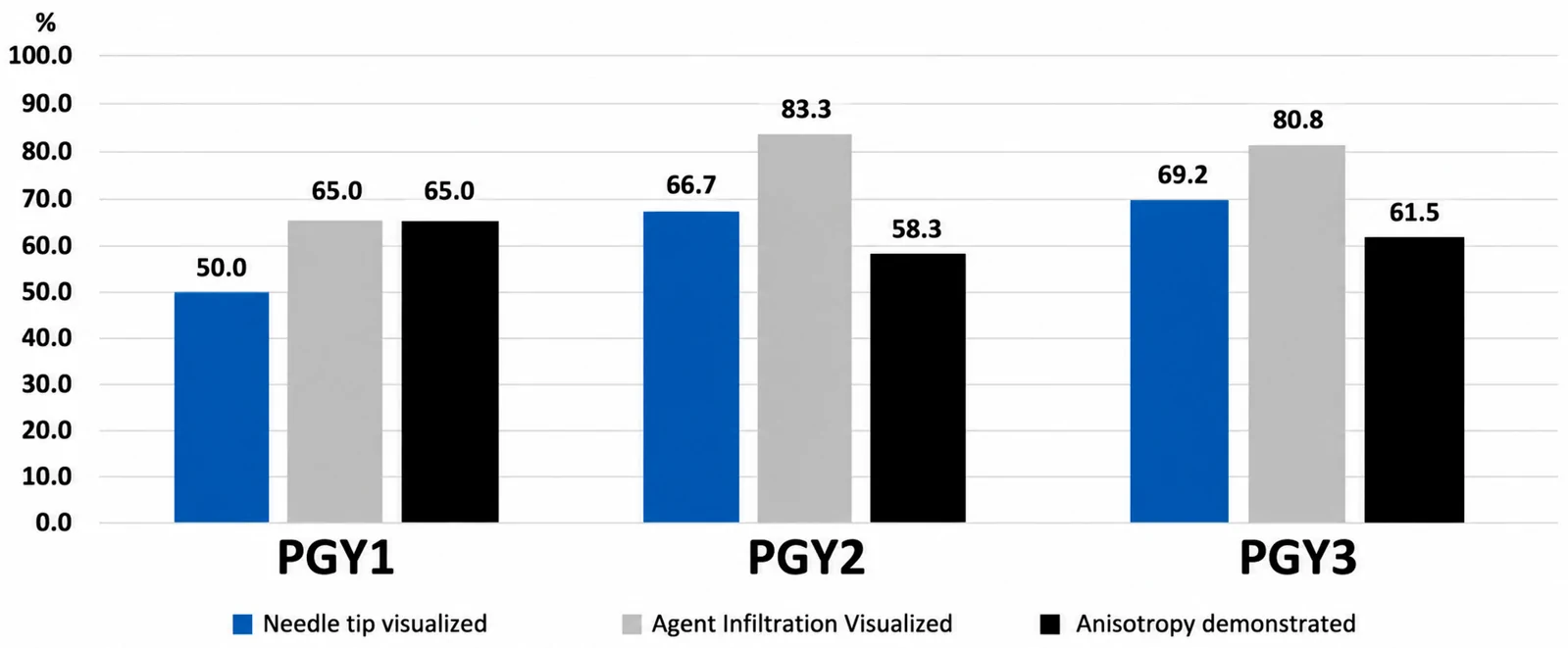
Technical performance measures by postgraduate year (PGY) (data are presented descriptively; no statistical comparisons between PGY groups were performed).

To further describe technical performance, ultrasound-related outcomes were stratified by operator training level from postgraduate year 1 (PGY-1) through postgraduate year 3 (PGY-3). Descriptive differences in needle-tip visualization, anesthetic-infiltration visualization, and anisotropy were observed across PGY groups (Figure 3). Because no statistical comparisons between training levels were performed, these findings should be considered descriptive and should not be interpreted as demonstrating an association between level of training and technical performance. Nevertheless, peripheral nerve ultrasound interpretation is itself operator-dependent, and recent evidence suggests that evaluator experience influences fascicle recognition and the accuracy of peripheral nerve ultrasound interpretation [22]. Future prospective studies with larger sample sizes may better evaluate differences in ultrasound proficiency across levels of operator training. In the future, a multi-study, prospective trial may better demonstrate differences in skill levels and ultrasound proficiency across various levels of operator training and skills.

### Study Limitations

This study faced multiple limitations in the way it was carried out and in its study design. Primary limitations included its two-hospital, regionally limited design. This in turn also contributed to its limited population size. There is a strong likelihood that other nerve blocks, including ultrasound-assisted and landmark-based nerve blocks, were completed in the ED that may not have been adequately documented in the Q-Path system, thus introducing selection bias. It is possible that ineffective nerve blocks, those requiring multiple attempts or site change, or those that were aborted due to complications may not have been documented or saved in the Q-Path system.

Another limitation was the lack of standardized clinical documentation following nerve block performance. In particular, pain scores and other measures of clinical response were inconsistently documented, preventing reliable assessment of analgesic effectiveness. Therefore, clinical effectiveness was not evaluated as an outcome in this study. Future prospective studies incorporating standardized pre- and post-procedure pain assessments and predefined clinical outcomes are needed to evaluate the effectiveness of trainee-performed nerve blocks.

Lastly, this study found significant difficulty in demonstrating long-term effects and complications of these nerve blocks. As previously stated, documentation was often incomplete, which led to adverse reactions or oligoanalgesia being poorly monitored. Additionally, as most of these patients had traumatic orthopedic injuries, inpatient medicine teams often monitored these patients before and after surgery and may not be experienced with the presentation of post-block complications. Finally, patients who were discharged were often lost to follow-up or did not have documented comments regarding their nerve blocks in post-block clinical visits. While the literature has been clear that post-block complications, such as local anesthetic systemic toxicity syndrome, ischemia, and direct nerve injury, are rare, the inability to monitor these effects from blocks performed may lead to confounding when evaluating nerve blocks completed by resident physicians in the acute setting. Future research, such as prospective study designs with specific follow-up and standardized documentation, may remedy these concerns.

## 5. Conclusions

In this retrospective cohort of archived ultrasound-guided peripheral nerve blocks performed by Emergency Medicine trainees, technical performance, including needle-tip visualization and visualization of anesthetic infiltration, varied across procedures and levels of training, and few complications were recorded. These findings provide descriptive data regarding trainee performance of ultrasound-guided regional anesthesia in the ED. Because the study was limited to archived procedures and may not have captured unsuccessful, aborted, or undocumented blocks, the findings should not be interpreted as establishing the overall safety or clinical effectiveness of trainee-performed nerve blocks. Prospective studies with standardized clinical outcomes, documentation, and systematic follow-up are needed to more definitively evaluate procedural success and safety.

## Data Availability

The original contributions presented in this study are included in the article. Further inquiries can be directed to the corresponding author.
